# Activation of AMPK/SIRT1 axis is required for adiponectin-mediated preconditioning on myocardial ischemia-reperfusion (I/R) injury in rats

**DOI:** 10.1371/journal.pone.0210654

**Published:** 2019-01-17

**Authors:** Maria Assunta Potenza, Luca Sgarra, Carmela Nacci, Valentina Leo, Maria Antonietta De Salvia, Monica Montagnani

**Affiliations:** 1 Department of Biomedical Sciences and Human Oncology-Pharmacology Section, Medical School-University of Bari "Aldo Moro", Bari, Italy; 2 Department of Emergency and Organ Transplantation-Section of Cardiovascular Diseases, Medical School-University of Bari "Aldo Moro", Bari, Italy; Indiana University School of Medicine, UNITED STATES

## Abstract

**Background:**

Adiponectin (AD) cardioprotective activities are mediated by AMPK, a fuel-sensing molecule sharing common targets and cellular activities with SIRT-1. Whether AD preconditioning may involve SIRT-1 activity is not known; however, the protective role of SIRT-1 during ischemia and the potential interplay between AMPK and SIRT-1 suggest this possibility.

**Methods:**

Isolated hearts from male Sprague-Dawley rats (n = 85) underwent ischemia/reperfusion (I/R, 30/180 min). Preconditioning with resveratrol (RSV, SIRT-1 activator) was compared to preconditioning with AD alone, or in combination with compound C (CC, AMPK inhibitor) or sirtinol (STN, SIRT-1 inhibitor). For each heart, left ventricular end-diastolic pressure (LVEDP), left ventricular developed pressure (dLVP), coronary flow (CF) and left ventricular infarct mass (IM) were measured, together with the phosphorylation/activation status of AMPK, LKB1, eNOS and SIRT-1, at the beginning (15 min) and at the end (180 min) of reperfusion.

**Results and conclusions:**

When compared to I/R, both RSV and AD improved cardiac function and reduced IM (p < 0.01, p < 0.05, respectively). Cardioprotective effects of AD were completely reversed in the AD+CC group, and significantly attenuated in the AD+STN group. Both RSV and AD increased eNOS, AMPK and LKB1 phosphorylation (for each parameter: p < 0.05 vs. I/R, in both RSV and AD treatment groups) at 15 min of reperfusion, and SIRT-1 activity at the end of reperfusion (p < 0.01, p < 0.05 vs. I/R, respectively). Interestingly, AD-mediated phosphorylation of AMPK and LKB1, and SIRT-1 deacetylation activity was markedly reduced in both the AD+CC and AD+STN groups (p < 0.05 vs. AD). Thus, AD-mediated cardioprotection requires both AMPK and SIRT-1 signaling pathways, that act as a component of a cycle and regulate each other’s activities.

## Introduction

The extension of the myocardial infarct size resulting from both ischemia and reperfusion (I/R) injury is a key determinant for the prognosis of patients who survive an acute myocardial infarction (MI) [1]. The molecular plasticity of the heart to adapt to I/R stress has encouraged the exploration of conditioning strategies (known as pre-and post-conditioning) that may enhance cardioprotection by facilitating adaptative responses and ameliorating the heart functional recovery [2]. However, and despite the large number of signaling pathways identified so far, pharmacologic approaches to attenuate the consequences of I/R injury are still of limited efficacy and fail to translate into useful clinical treatments. In part, this may be due to specific alterations in fundamental cellular signaling cascades that the same risk factors predisposing to the development of ischemia (e.g., atherosclerosis, hypertension, diabetes) play in the response to cardioprotective interventions [3]. Thus, investigation on cellular mechanisms ensuring cardioprotective effect from I/R injury under specific conditions is still ongoing.

During ischemia [4], the reduced oxygen availability causes intracellular acidosis and impairs cardiac energy metabolism by increasing levels of inorganic phosphates, resulting in the unbalance of both AMP/ATP and NAD/NADH ratio [5]. These metabolic changes affect, among others, the AMP-activated protein kinase (AMPK) [6] and the NAD^+^-dependent deacetylase sirtuin-1 (SIRT-1) [7], two fuel-sensing molecules sharing common intracellular targets [8–10], and regulating processes as survival and cellular longevity [11], cellular energy and metabolism [12], inflammation [13], apoptosis and ROS reduction [14], mitochondrial biogenesis and function [15].

The critical relevance of AMPK in cardioprotection has been persistently demonstrated by the severe heart contractile dysfunction under AMPK inactivation or deletion [16,6,17]. AMPK promotes cell survival in ischemic heart by inducing cardiomyocyte autophagy, by attenuating ER stress, and by decreasing myocardial oxygen consumption via endothelial nitric oxide synthase (eNOS)-mediated NO production [18–20].

A number of studies suggest that SIRT-1, as AMPK, is activated in response to changes in nutrient availability [21,22] and energy expenditure [12,23]. In mouse skeletal muscle, SIRT-1 activity is enhanced by AMPK, that increases cellular NAD^+^ levels and NAD/NADH ratio and favours deacetylation of SIRT-1 downstream targets [23]. Interestingly, Liver Kinase B1 (LKB1), a key upstream activator of AMPK, has been identified among SIRT-1 deacetylation targets [24], and SIRT-1-dependent dysregulation of the LKB1-AMPK pathway has been implied in the development of dysmetabolic conditions. In the heart, SIRT-1 protects cardiomyocytes against oxidative stress, apoptosis, and aging [25,26] and cardiac SIRT-1 overexpression limits the extent of infarct size during I/R injury [27]. Coherently, genetic or pharmacological abrogation of SIRT-1 reduces the beneficial effects of ischemic preconditioning in hearts subjected to I/R injury [28–30].

Heart AMPK may be activated by several mediators including adiponectin (AD), whose circulating levels are markedly reduced under obesity, diabetes and insulin-resistance conditions, as well as in patients with hypertension, coronary artery disease and myocardial infarction [31–33]. Beside anti-inflammatory [34,35], antiatherogenic ad vasorelaxant properties promoted by stimulation of eNOS [36,37], AD exerts direct cardioprotective effects mediated by signaling cascades involving AMPK and LKB1 [38]. In rat hearts subjected to I/R injury, activation of the AMPK/AKT/eNOS pathway by exogenous AD limits the contractile dysfunction and decreases the infarct size both in preconditioning [39] as well as in postconditioning administration [40]. Accordingly, AD-knockout mice demonstrate impaired AMPK activation during ischemia and increased susceptibility to ischemic injury [41], reverted by adenoviral-mediated AD expression [41]. Whether cardioprotection induced by exogenous AD administration may involve SIRT-1 activity is not known; however, the protective role of SIRT-1 during ischemia and the potential interplay between AMPK and SIRT-1 suggest this possibility. Therefore, this study was planned to investigate the hypothesis that AD-mediated preconditioning may protect rat hearts subjected to I/R injury via activation of the AMPK/SIRT1 axis.

## Materials and methods

All procedures in animals were approved by the Committee on the Ethics of Animal Experiments of the University of Bari and performed under Authorization for the Use of Laboratory Animals of Ministry of Health (Italian Government, prot.n. 216/2016-PR).

### Animals

A total of 85 adult male Sprague-Dawley (SD) rats (Envigo, Udine-Italy) weighing 250–300 g were housed in a temperature-, humidity- and light-controlled room of the Department animal facility. Rats were randomly assigned to treatments illustrated under “Experimental protocol”, anesthetized with sodium pentobarbital (80 mg/kg body weight i.p.), heparinized (400 UI/100 g body weight i.p.) and euthanized by cervical dislocation. All efforts were made to minimize animal suffering.

Hearts from SD rats were isolated and mounted on a Langendorff perfusion system (Radnoti LLC, USA), as previously described [42]. Briefly, excised hearts were immediately subjected to aortic cannulation and perfused with modified Krebs-Henseleit solution (composed of (mmol/l): 118.5 NaCl; 4.7 KCl; 1.2 MgSO_4_; 1.2 KH_2_PO_4_; 1.25 CaCl_2_(H_2_O); 25 NaHCO_3_; 11 glucose) continuously gassed with a mixture of 95% O_2_ and 5% CO_2_ (pH 7.4) at 37°C. The perfusion pressure (PP) was kept constant at 80 mmHg for the whole experimental procedure.

Hemodynamic parameters evaluated during reperfusion included isovolumetric recordings of left ventricular systolic (LVSP) and end-diastolic (LVEDP) pressures obtained from a balloon catheter inserted into the left ventricle through the auricle (LVEDP set to 5–10 mmHg at the beginning of the stabilization period). Coronary flow was measured by timed collection of the coronary effluent. Left ventricular developed pressure (dLVP) was calculated as dLVP = LVSP—LVEDP. The rate of pressure product (RPP) was not included among functional parameters measuring post-ischemic heart recovery: although representing an important index of cardiac effort, RPP may indicate oxygen consumption and myocardial workload only in the presence of a positive force–frequency relationship. However, isolated rodent hearts demonstrate a negative force–frequency relationship [43], and the potential significance of RPP in the Langendorff-perfused isolated rat heart is uncertain.

All data were acquired at a sampling rate of 1 kHz by a 4-channel PowerLab system (ADInstruments, UK) and analyzed using LabChart 7 Pro Software (ADInstruments, UK). A side arm attached to a chamber in connection to the aortic cannula was used for drugs administration at the onset of ischemia.

### Experimental protocol

Each heart (10 hearts/group) was allowed to stabilize for 20 min, and then subjected to 30 min of global no-flow ischemia followed by 180 min of reperfusion. Only hearts with LVSP between 60–160 mmHg and coronary flow 8–16 ml/min were studied. Thus, in each group, the number of hearts studied indicates the difference between the initial number of hearts assigned and the number of hearts excluded (10 –x/ group). Pharmacological preconditioning was obtained by bolus infusion of drugs (3 mL/1 min), alone or in combination, directly into the aortic cannula at the onset (during the first minute) of ischemia. According to specific protocols, the hearts were randomly exposed to the following treatments: ischemia/reperfusion (I/R) (vehicle-treated; n = 7), human recombinant adiponectin (AD) (Adipogen; 3 μg/mL; n = 6), resveratrol (RSV) (Sigma-Aldrich, 10 μM; n = 8), sirtinol (STN) (Sigma-Aldrich, 10 μM; n = 6); compound C (CC) (Sigma-Aldrich, 10 μM; n = 5), AD+STN (n = 6), and AD+CC (n = 6). For WB experiments on signaling pathways activation during the early reperfusion time (15 min), 3 additional hearts/group were used for I/R, AD, RSV, AD+STN and AD+CC treatments.

Stock solutions of RSV (50 mM), STN (1 mM) and CC (12,5 mM) were prepared in pure DMSO. Final dilutions of these drugs were prepared in modified Krebs-Henseleit solution immediately before use and contained DMSO 0.1%, according to recommendation related to potential toxic effects for DMSO concentrations > 1% [44]. AD was directly dissolved in modified Krebs-Henseleit solution. Recombinant human AD shows overlapping biological activity among human and rodent species [45]. Haemodynamic parameters and coronary flow were recorded twice before ischemia, and after 5, 15, 30, 45, 60, 90, 120 and 180 min of reperfusion.

### Determination of area at risk and infarct size

At the end of reperfusion period, hearts were incubated in freshly prepared 2,3,5 triphenyltetrazolium chloride (TTC 1% w/v phosphate buffer pH 7.4, 37° C, 20 min) and then weighed and frozen (–80° C, 30 min). Each heart was subsequently sliced in transverse sections (approximately 1.5 mm thick), and each slice weighed and scanned on both sides by a flat-bed scanner (Epson 3490). The infarct area on each color image (TTC unstained) was traced in a blind fashion and measured by planimetry (Image-Tool 2.0 Software NIH, USA). Area at risk (AAR, representing total infarct area with respect to total muscle mass) was calculated by the sum of individual slice weights according to the following formula: (*AIn/AARn*) x (*Wn/Wtotal*), where AI is the infarct area of each slice (n = 7), Wn is the weight of the respective section (n) and Wtotal is the sum of all slice weights [46].

### Tissue processing

*Western Blot analysis*–In another set of experiments, hearts (n = 3/group) were assigned randomly to pharmacological treatments previously described, and subjected to I/R injury. At the beginning of reperfusion period (15 min), samples of the left ventricular tissue were freeze-clamped in liquid nitrogen and then stored at -80° C until further analysis. Frozen samples were homogenized on ice in cold RIPA lysis buffer containing 1% Nonidet P-40, 0.5% Sodium deoxycholate, 0.1% SDS, 50 KIU aprotinin, 100 mM sodium orthovanadate, 10 mg/ml PMSF, and then centrifuged at 4° C for 15 min at 13.000 g. Protein level was determined by Bradford’s method [47]. Equal amounts of proteins (100 μg) were separated by 10% SDS-PAGE and subjected to immunoblotting with the following primary antibodies (dilution 1:1000): eNOS, (Transduction Laboratories, Lexington KY), Ph-eNOS, AMPK, Ph-AMPK, LKB1, Ph-LKB1, Ph-SIRT-1 (Cell Signaling Technology, MA), SIRT-1 (Santa Cruz Biotechnology, Inc, CA). Incubation with HRP-linked antimouse or antirabbit secondary antibodies (Santa Cruz Biotechnology Inc., CA) (1:3000) was performed for 1 h at room temperature. Immunoblotting results were visualized by Molecular Imager ChemiDoc XRS System (Bio-Rad Laboratories, CA). Images were captured with QuantityOne Software (Bio-Rad Laboratories, CA) and blots quantified by scanning densitometry (ImageJ, NIH, Bethesda, MD).

*SIRT-1 activity (deacetylation) assay—*For SIRT-1 deacetylation assay, at the end of reperfusion period (180 min), samples of the left ventricular tissue (the tip from hearts of each group) were freeze-clamped in liquid nitrogen and then stored at -80° C until further analysis. SIRT-1 deacetylation activity *in vitro* was determined by fluorimetric analysis with a specific fluorescent kit (Enzo Life Sciences, Italy), according to manufacturer’s instructions. This assay uses a small lysine-acetylated peptide comprising amino acids 379–382 of human p53, as substrate. The assay’s fluorescence signal is generated in proportion to the amount of deacetylation of the lysine corresponding to Lys-382, a known *in vivo* target of SIRT-1 activity, and this process is dependent on the addition of exogenous NAD^+^. Briefly, heart tissue (50–100 mg) was homogenized, and 25 μL homogenate incubated with 15 μL Fluor de Lys-SIRT-1 substrate (50 μM) and NAD^+^ (100 μM) for 60 min at 37° C. The reaction was stopped by adding a solution containing Fluor de Lys Developer and 2 mmol/L nicotinamide, and the fluorescence was monitored at 360 nm (excitation) and 460 nm (emission). Changes in fluorescence, measured as arbitrary fluorescence units (AFU) per min, was normalized to the amount of total protein in each sample (AFU/min/μg protein).

### Statistical analysis

A power analysis was prospectively conducted to determine the number of rats needed. Based on coefficients of variation calculated for similar studies, a sample size of 5 (in each group) was sufficient to detect 10% differences in infarct area extent (α = 0.05) with a power of 0.80. All data are expressed as mean ± standard error (SE) of *n* experiments (*n* = number of rats). For evaluation of functional parameters, statistical significance between groups was measured by two-way repeated measures ANOVA (analyzing effect of time, group and time by group interaction), followed by Tukey test for multiple comparison, as appropriate. For infarct mass, SIRT-1 deacetylation activity and immunoblotting experiments, results between groups were compared by one-factor ANOVA followed by Bonferroni correction. Values of p < 0.05 were considered to indicate statistical significance. All analysis was performed using Statistica Release 7 (Statsoft Institute Inc).

## Results and discussion

Ischemic preconditioning, although effective in experimental studies, has limited clinical applicability. Therefore, pharmacological treatments that may reproduce the protective effects of this approach have been regarded as a more feasible strategy against acute ischemic insult [2,48–51]. However, assessment of any potential benefit from pharmacological preconditioning must preliminary exclude whichever protective effect exerted by the manoeuvre of conditioning *per se*. Thus, before evaluating the mechanisms involved in AD-mediated cardioprotection, hearts exposed to 30 min ischemia followed by 180 min reperfusion (I/R group) were first compared with hearts infused with vehicle alone (3 mL/1 min) at the onset of ischemia; both functional parameters and the extent of infarct area did not significantly differ between hearts from these two groups (S1 Fig). Therefore, in all subsequent experiments, the effect of each individual treatment was compared with data obtained in the I/R group. As shown in Table 1, baseline functional parameters from isolated rat hearts subjected to distinct pharmacological treatments were not significantly different.

**Table 1:** General and hemodynamic parameters at baseline.

Groups	Body Weight (g)	Heart Weight (g)	dLVP (mmHg)	LVEDP (mmHg)	Heart Rate (BPM)	Flow Rate (ml/min)
**I/R**	380 ± 10	1.02 ± 0.4	86.5 ± 2.3	11.6 ± 2	265 ± 29	12 ± 5
**RSV**	378 ± 11	0.98 ± 0.2	84.9 ± 8.1	9.5 ± 5	275 ± 31	13 ± 2
**AD**	384 ± 12	0.93 ± 0.5	92.2 ± 4.4	11.1 ± 2	279 ± 19	11 ± 3
**STN**	390 ± 10	0.99 ± 0.2	92.2 ± 9.9	10.5 ± 2	279 ± 29	12 ± 3
**CC**	385 ± 11	0.97 ± 0.1	99.5 ± 9.9	11.4 ± 2	273 ± 24	11 ± 3
**AD + CC**	392 ± 13	0.95 ± 0.4	129.3 ± 20.2	13.7 ± 1	265 ± 30	11 ± 4
**AD + STN**	385 ± 14	0.94 ± 0.1	98.3 ± 14.1	12.3 ± 2	269 ± 27	11 ± 2

Values represent the mean ± SD for values measured during the last 7 min of stabilization prior to ischemia. *dLVP* developed left ventricular pressure, *LVEDP* left ventricular end-diastolic pressure. No significant difference was found between groups (one-way ANOVA test).

### AD preconditioning improves post-ischemic recovery of ventricular function in a AMPK and SIRT-1-dependent fashion

To investigate the involvement of AMPK/SIRT-1 on AD preconditioning, functional parameters of left ventricular function obtained in hearts exposed to AD were compared with those achieved in hearts infused with RSV, known to activate SIRT-1 and used here as positive control. Previous studies have shown that RSV, a natural polyphenol found in red wine and grapes, is able to improve post-ischemic cardiac function by enhancing the anti-oxidative capacity of the heart [52]: these beneficial effects have been ascribed, at least in part, to activation of SIRT-1-dependent transcriptional regulatory mechanisms [26,53] resulting in upregulated AMPK expression and improved cardiac retrieval [54].

**Fig 1:**
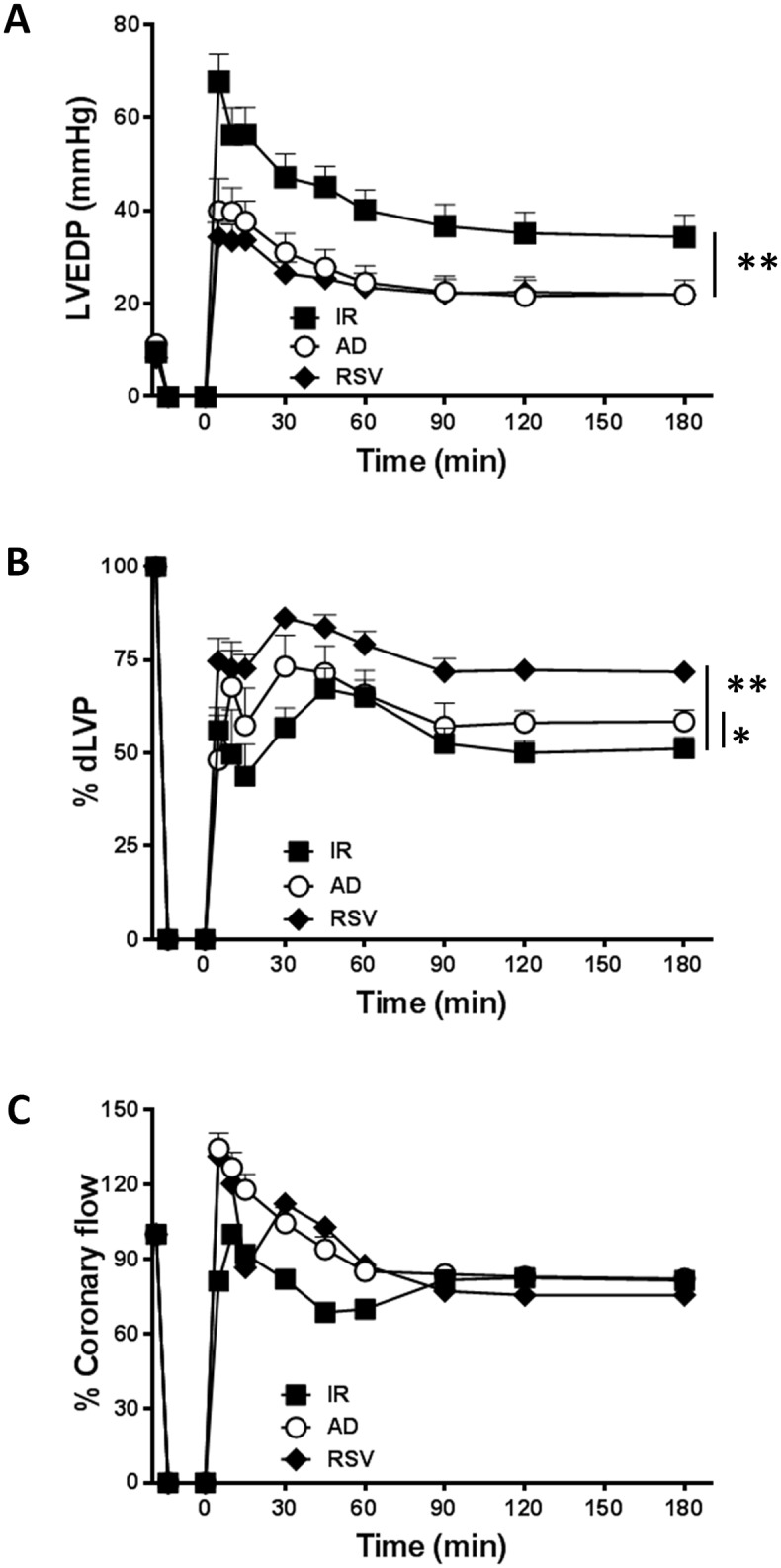
AD preconditioning improves post-ischemic recovery of ventricular function with effects partially overlapping those induced by RSV preconditioning. Hearts preconditioned with adiponectin (AD) were compared to hearts exposed to vehicle-administration (I/R) or resveratrol (RSV), used as a positive control of SIRT-1 activation. **A. *Diastolic function*.** Left ventricular end diastolic pressure (LVEDP, mmHg) during the 180 min of reperfusion following 30 min of global ischemia (**p < 0.01 *vs*. I/R). **B. *Systolic function***. Percent variation of developed left ventricular pressure (dLVP) for each group during the 180 min of reperfusion, respectively (*p < 0.05 AD *vs*. I/R, **p < 0.01 RSV *vs*. I/R) **C.** Percent variation of ***coronary flow*** during the 180 min of reperfusion. Two-way repeated measures ANOVA was employed to determine the main effect of time, group and time by group interaction.

During the whole reperfusion time (180 min), LVEDP, an index of left ventricular contractility whose values increase proportionally to the degree of ventricular failure, was significantly lower in hearts from RSV group when compared to I/R group (**p < 0.01, Fig 1A). Concomitantly, systolic left ventricular pressure (LVP max), indicating post-ischemic systolic functional recovery, was substantially higher (p < 0.01 *vs*. I/R group, S2 Fig). Accordingly, the post-ischemic dLVP, which summarizes systolic and diastolic pressure values, was significantly improved in RSV group (**p < 0.01 *vs*. I/R, Fig 1B). No changes were observed in coronary flow between hearts exposed to RVS or I/R (Fig 1C).

Consistent with previous studies reporting the protective effects of AD in rodent hearts [39,41], AD preconditioning decreased LVEDP during the whole reperfusion period, with LVEDP values substantially overlapping those obtained in hearts exposed to RSV preconditioning (**p < 0.01 *vs*. I/R, Fig 1A); AD preconditioning also significantly improved dLVP with respect to I/R group (*p < 0.05 *vs*. I/R, Fig 1B*)*, although dLVP in the AD group and RSV group did not completely overlie (*p < 0.05 *vs*. RSV, Fig 1B*)*. Interestingly, during the first 60 min of reperfusion, coronary flow tended to increase in AD group with respect to I/R group (Fig 1C), though this effect did not reach statistical significance.

Taken together, when compared to hearts exposed to I/R alone, parameters of left ventricular function were significantly ameliorated in hearts subjected to preconditioning with AD or RVS, thus supporting the hypothesis that both these compounds might activate similar pathways.

**Fig 2:**
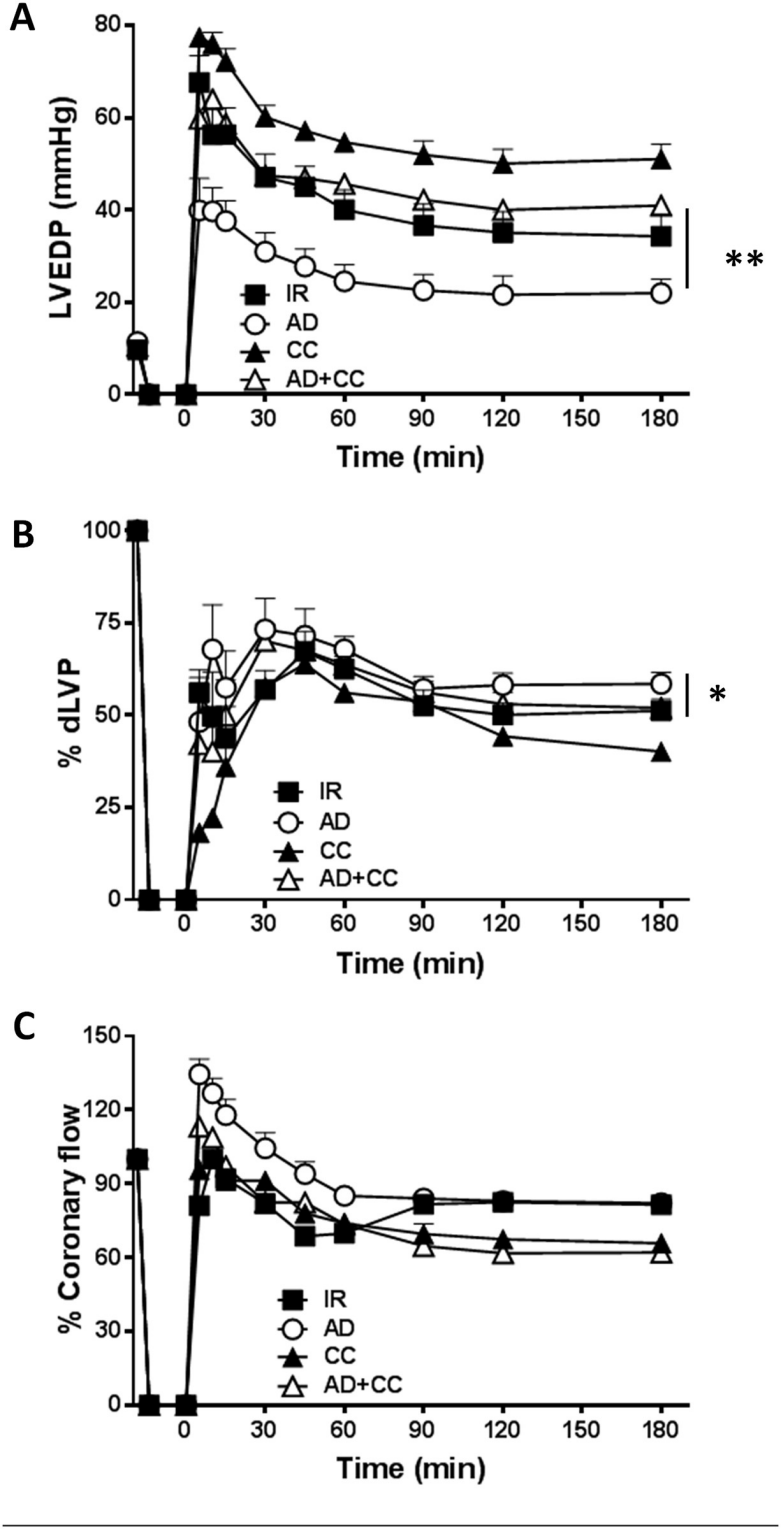
The AD-mediated post-ischemic left ventricular recovery depends on AMPK activation. Hearts preconditioned with AD were compared to hearts infused with compound C, a specific inhibitor of AMPK, alone (CC), or in combination with AD (AD + CC). **A**. ***Diastolic function*.** Left ventricular end diastolic pressure (LVEDP, mmHg) during the 180 min of reperfusion (**p < 0.01 AD *vs* AD + CC) **B**. ***Systolic function*.** Percent variation of developed left ventricular pressure (dLVP) for each group during the 180 min of reperfusion (*p < 0.05 AD *vs* AD + CC). **C.** Percent variation of ***coronary flow*** during the 180 min of reperfusion. Two-way repeated measures ANOVA was employed to determine the main effect of time, group and time by group interaction.

The involvement of AMPK in AD-mediated post-ischemic left ventricular recovery was verified by comparing results from hearts infused with AD alone and AD in combination with compound C (CC), a specific inhibitor of AMPK (Fig 2A–2C). As expected, preconditioning with CC alone did not improve (but rather worsened) parameters of the left ventricular function observed in hearts from I/R group (Fig 2A–2C).

In hearts concomitantly treated with AD+CC, values of LVEDP were significantly higher than in the AD group during the whole reperfusion interval (**p < 0.01), and similar to those measured in the I/R group (Fig 2A). On the other hand, values of dLVP obtained under AD preconditioning were decreased in the AD+CC group (*p < 0.05 *vs*. AD group, Fig 2B); moreover, the increased coronary flow observed in the AD group during the first 60 min of reperfusion was lost in the AD+CC group (Fig 2C). These results reinforce the importance of AMPK activity in AD-mediated effects, consistent with the key role of AMPK in modulating energy homeostasis of isolated cardiomyocytes [55] and whole heart [56,57] in response to ischemic stress [16] and cardiac hypertrophy [58].

**Fig 3:**
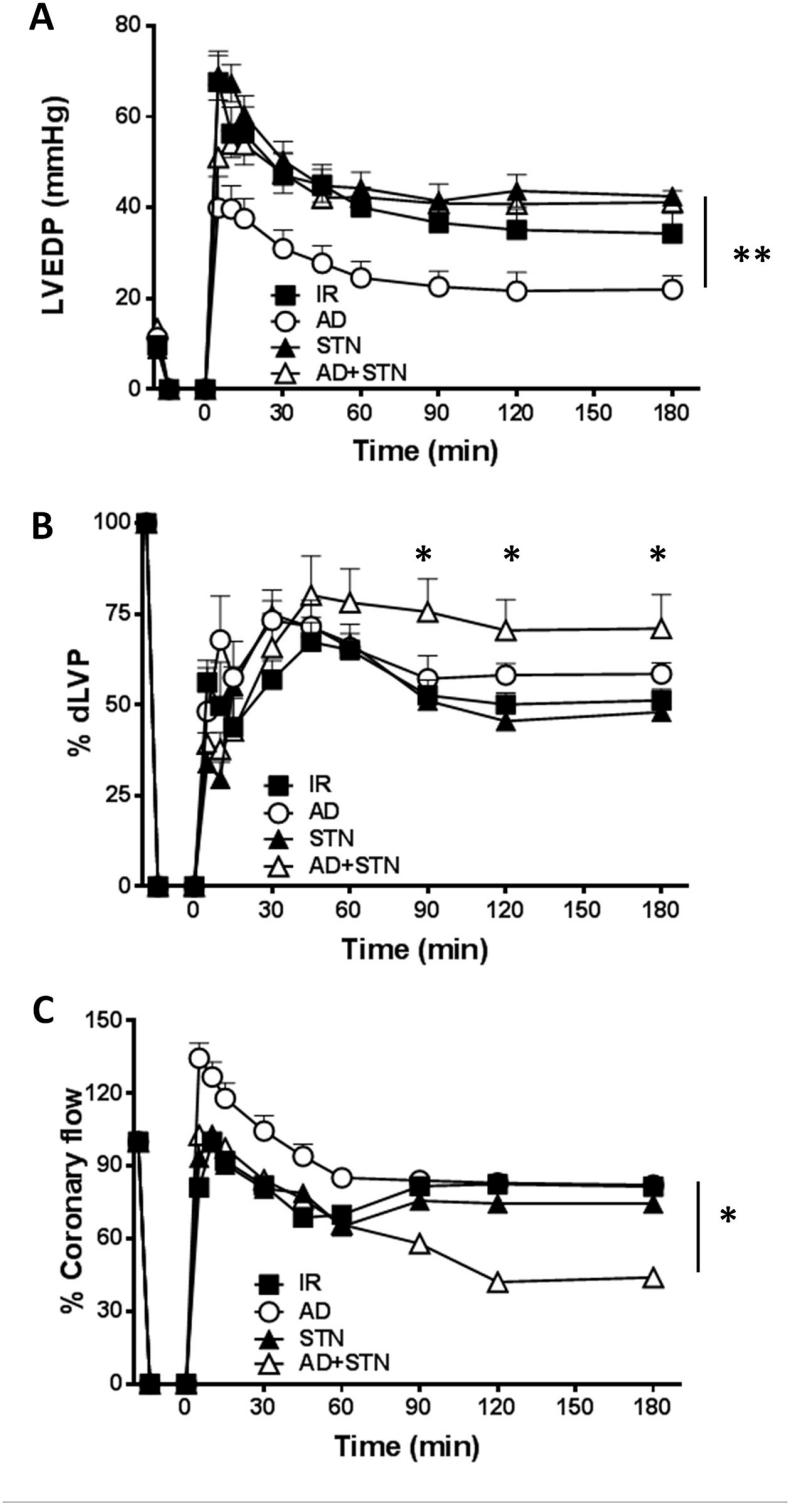
The AD-mediated post-ischemic left ventricular recovery partially depends on SIRT-1. Hearts preconditioned with AD were compared to hearts infused with sirtinol, a specific inhibitor of SIRT-1, alone (STN), or in combination with AD (AD + STN). **A**. ***Diastolic function*.** Left ventricular end diastolic pressure (LVEDP, mmHg) during the 180 min of reperfusion (**p < 0.01 AD *vs* AD + STN); **B**. ***Systolic function*.** Percent variation of developed left ventricular pressure (dLVP) for each group during the 180 min of reperfusion (*p < 0.05 at 90, 120, 180 min AD + ST *vs* I/R group). **C.** Percent variation of ***coronary flow*** during the 180 min of reperfusion (*p < 0.05 AD *vs*. AD + STN group). Two-way repeated measures ANOVA was employed to determine the main effect of time, group and time by group interaction.

To evaluate the potential contribution of SIRT-1 in AD-mediated preconditioning, functional parameters of post-ischemic recovery obtained in hearts treated with AD alone were compared with those exposed to AD in combination with STN, a well-known inhibitor of NAD-dependent histone deacetylase SIRT-1. Exposure to STN alone did not modify LVDEP, dLVP or coronary flow with respect to I/R group (Fig 3A–3C). On the other hand, in hearts subjected to AD+STN preconditioning, values of LVEDP during whole reperfusion period were significantly higher with respect to LVEDP observed in hearts receiving AD alone (**p < 0.01, Fig 3A). Surprisingly, LVP max, indicating post-ischemic systolic functional recovery, was slightly increased in AD+STN group during the second and third hour of reperfusion (p < 0.05 at 90, 120, 180 min vs. respective values in the I/R and AD groups, S2 Fig). As a consequence, at the same time-intervals, dLVP values obtained under concomitant administration of AD+STN were higher than in the I/R group (p < 0.05 at 90, 120, 180 min vs. respective values, Fig 3B), and did not significantly differ from the AD group (Fig 3B).

On the other hand, coronary flow was reduced during the second and third hour of reperfusion in hearts receiving AD+STN (*p < 0.05 *vs*. AD group, Fig 3C). Taken together, these findings suggest that inhibition of SIRT-1 by STN modifies only partially the AD-mediated effects, with a more pronounced effect on global left ventricular recovery than on isolated systolic function, and a negative effect on coronary perfusion.

### Inhibition of either AMPK or SIRT-1 activity abrogates the beneficial effects of AD-mediated preconditioning on infarct mass extension

**Fig 4:**
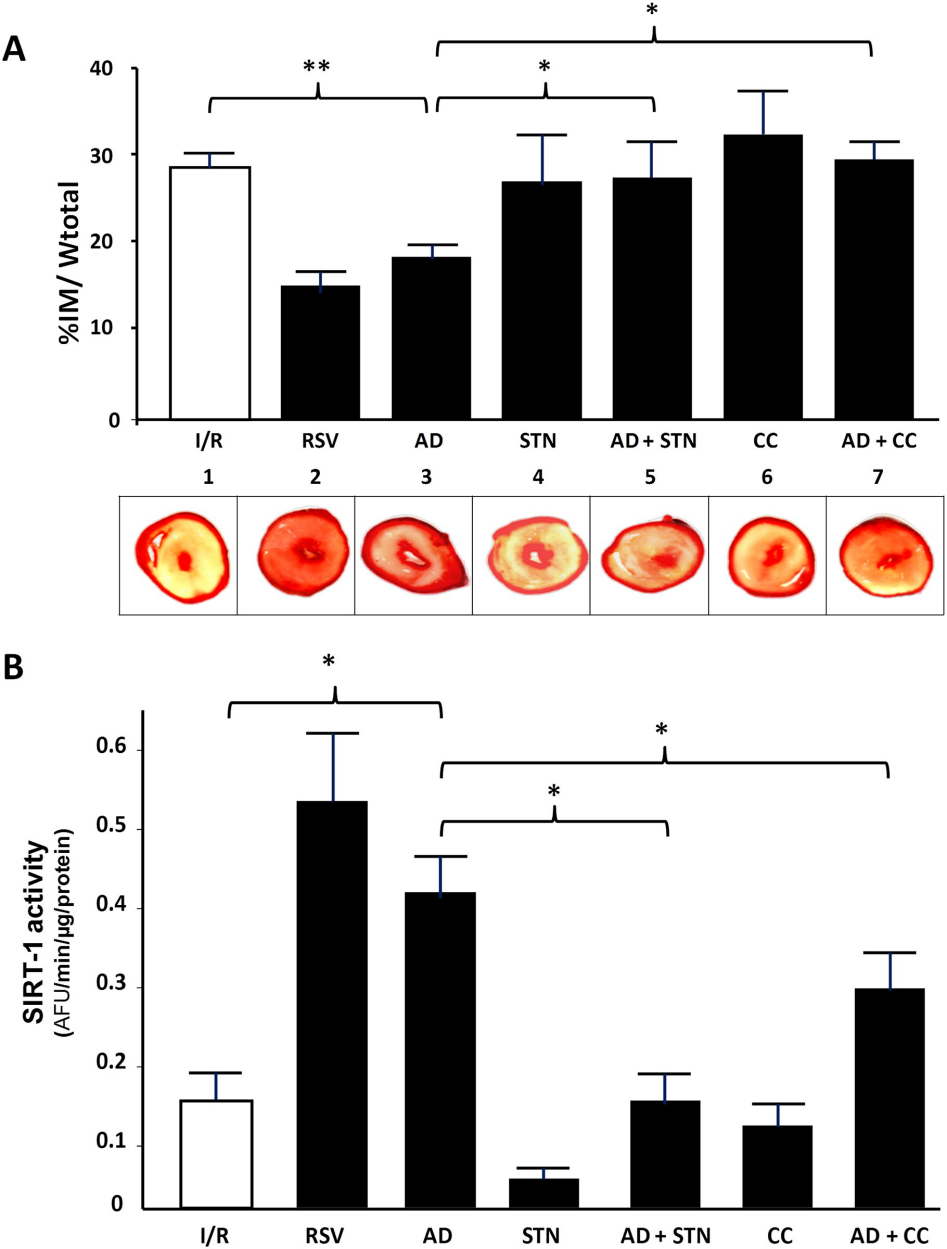
Inhibition of either AMPK or SIRT-1 activity abrogates AD-mediated preconditioning on infarct mass extension. **A. Upper panel.** Quantification of necrotic tissue, expressed as percentage of the left ventricular mass, in hearts exposed to various treatments at the end of the reperfusion interval. Statistical differences between groups were evaluated by one-factor ANOVA followed by Bonferroni correction. Values are expressed as mean ± SEM (**p < 0.01; *p < 0.05). **A. Lower panel.** Representative images of left ventricular sections quantified in panel above are shown. Hearts were cut into transverse slices and stained with TTC to differentiate necrotic (unstained) from viable (red) myocardium. **B.** SIRT-1 activity measured as arbitrary fluorescence units (AFU) per min, normalized to the amount of total protein in each sample (AFU/min/μg protein), in hearts exposed to various treatments at the end of the reperfusion interval. Statistical significance between groups was evaluated by one-factor ANOVA followed by Bonferroni correction. Values are expressed as mean ± SEM (*p < 0.05. **p < 0.01).

Besides functional parameters, the most reliable evaluation of the effectiveness for a cardioprotective strategy is based on the extent of the infarct area. Thus, at the end of the reperfusion interval, the percent of ischemic area was compared among hearts exposed to various treatments. Consistent with the improved cardiac performance suggested by parameters of left ventricular function, the infarct size was significantly smaller in hearts exposed to both RVS or AD preconditioning when compared to I/R group (16 ± 4.1% and 19.6 ± 1.4%, respectively, *vs*. 28.1 ± 1.1%; p < 0.01) (Fig 4A). As expected, preconditioning with both STN or CC alone did not significantly reduce the extent of infarct area with respect to hearts exposed to I/R (p = 0.9; p = 0.79, respectively) (Fig 4A). Interestingly, the cardioprotective effects of AD preconditioning were lost in hearts concomitantly infused with either AD+STN or AD+CC (p < 0.05 vs. AD group) (Fig 4A).

Concomitantly, SIRT-1 activity was evaluated by measuring the amount of Lys-382 deacetylation under each specific treatment (Fig 4, *panel B*). SIRT-1 requires NAD^+^ as a substrate for its enzymatic activity, and RSV administration at the onset of ischemia has been shown to increase NAD^+^ bioavailability and SIRT-1 function, to improve left ventricular functional recovery and reduce the infarct mass [59]. In line with the significant amelioration of post-ischemic recovery, SIRT-1 activity was significantly increased in heart homogenates exposed to RSV preconditioning (p < 0.01 vs. I/R group). Qualitatively similar results were obtained in hearts exposed to AD preconditioning, where SIRT-1 activity was approximately 3-times higher than under control conditions (*p < 0.05 vs. I/R group).

Unsurprisingly, SIRT-1 activity was unchanged in hearts treated with STN, as well as in hearts exposed to CC preconditioning, when compared to hearts of the I/R group. The concomitant treatment with AD+STN markedly reduced the AD-mediated activation of SIRT-1 (*p < 0.05 vs. AD group). Finally, a significant difference in SIRT-1 activity was also measured in hearts exposed to AD+CC with respect to AD alone (*p < 0.05). These findings strongly support the ability of AD to trigger SIRT-1 activity, and reinforce the hypothesis that the AD-dependent cardioprotection might be mediated, at least partially, via SIRT-1 signaling. Interestingly, results obtained in the AD + CC group suggest that, under AMPK inhibition, the AD ability to activate SIRT-1 is impaired; this implies that AMPK activation is required to achieve a full activation of SIRT-1, and further strengthens the existence of an AMPK/SIRT-1 cycle responsible for AD preconditioning.

### AD requires a different timing for complete activation of AMPK, LKB1, eNOS and SIRT-1 signaling pathways in ischemic hearts

**Fig 5:**
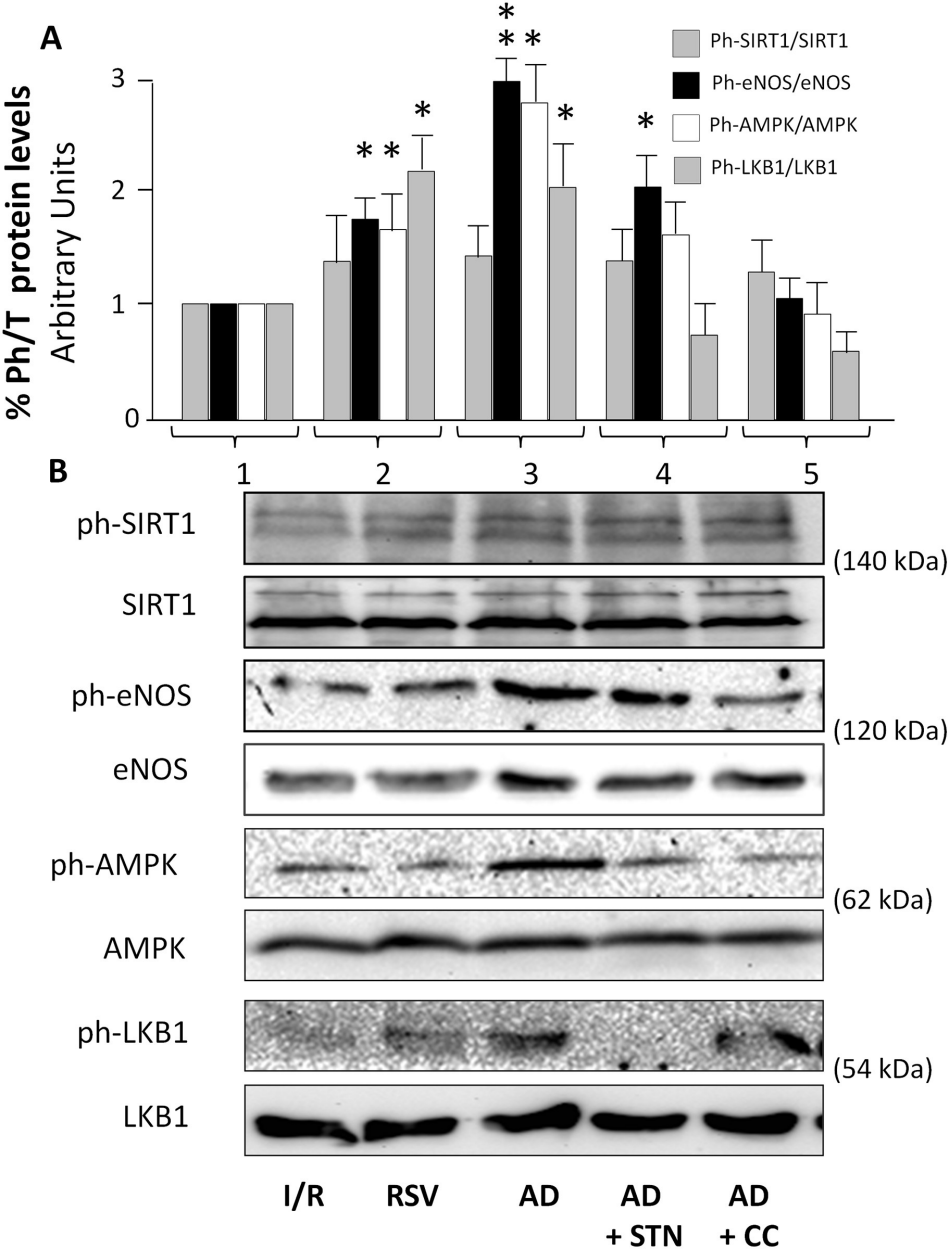
AD-mediated preconditioning activates AMPK, LKB1, and eNOS signaling pathways in the early post-ischemic reperfusion (15 min). Lysates from hearts exposed to 15 min of reperfusion were immunoblotted for phosphorylated and total isoforms of the indicated proteins. **A.** For each experimental condition, the ratio of phosphorylated/total SIRT-1, eNOS, AMPK and LKB1 proteins are expressed as the mean ± SEM of at least 3 independent experiments (each run in triplicate). Statistical differences between groups were evaluated by one-factor ANOVA followed by Bonferroni correction (*p< 0.05; **p < 0.01 *vs* I/R group). **B.** Representative immunoblots of total and phosphorylated SIRT-1, eNOS, AMPK and LKB1 proteins expression quantified in panel A are shown.

Although supportive of an AMPK/SIRT-1 involvement in AD-mediated cardioprotection, the observations described above do not provide direct evidence of a specific molecular activation, nor help to clarify whether AMPK and SIRT-1 are subsequently or concomitantly recruited by AD. Signaling cascades involved in intracellular events occurring immediately after exposure to the drug of interest were therefore evaluated 15 min after reperfusion. Total and phosphorylated protein levels of eNOS (at Ser 1177), SIRT-1 (at Ser 47), AMPK (at Thr172) and LKB1 (at Ser 428), an upstream kinase of AMPK and downstream target of SIRT-1, were compared in homogenates from hearts exposed to I/R, RSV and AD, alone and in combination with CC or STN (Fig 5).

Protein expression of SIRT-1, eNOS, AMPK and LKB1 did not vary among groups (Fig 5, *panel B*). In hearts treated with RSV (Fig 5, *column 2*), levels of phosphorylated eNOS, AMPK and LKB1 were increased with respect to I/R group (*p < 0.05); conversely, despite a weak increase, phosphorylated SIRT-1 was not significantly higher with respect to I/R group (p = 0.12; Fig 5, *column 1*). In homogenates of hearts subjected to AD preconditioning (Fig 5, *column 3*), SIRT-1 phosphorylation levels did not appear increased with respect to I/R group (p = 0.22); conversely, phosphorylation levels of eNOS, and of AMPK and LKB-1 were significantly higher (**p < 0.01, *p < 0.05, respectively) when compared to I/R group (Fig 5, *column 3*).

As expected, no change in SIRT-1 phosphorylation was detected in hearts concomitantly administered with AD+SNT (Fig 5, *column 4*); phosphorylation levels of eNOS were higher than in the I/R group (*p < 0.05), but reduced if compared to the AD group (*p < 0.05); both AMPK and LKB1 phosphorylated levels were similar in the AD+SNT and in the I/R groups, and lower than in the AD group (*p < 0.05). Finally, in the AD+CC group (Fig 5, *column 5*), administration of an AMPK inhibitor (CC) did not affect SIRT-1 phosphorylation, but completely reversed the ability of AD to increase phosphorylation of eNOS, AMPK and LKB1.

Collectively, these observations confirm that the AD-mediated cardioprotective effects require the early activation of both AMPK and LKB1, concurring to increase the AD-mediated eNOS phosphorylation. Although no significant variation in phospho-SIRT-1 was detected under these conditions, the impaired AMPK and LKB1 phosphorylation, along with the limited eNOS phosphorylation observed in the AD+STN group, suggest that SIRT-1 is required for full activation of the AD-mediated signaling pathways and subsequent cardioprotection. On this respect, it is important to take into account that SIRT-1 possesses at least 13 serine/threonine residues known to be target of distinct kinases (including LKB1, JNK and Cdk1), that SIRT-1 deacetylase activity increases proportionally to global phosphorylation levels, and that phosphorylation on specific sites increases the suitability of SIRT-1 as a substrate for additional kinases [60]. It is possible that SIRT-1 phosphorylation at Ser 47 might not be detectable at the early phase of post-ischemic reperfusion; however, and although SIRT-1 deacetylation activity was not directly measured 15 min after reperfusion, acetylation levels on NF-kB p65 (at Lys 310) as well as panacetylated protein levels were slightly decreased in both RSV and AD groups with respect to I/R group, suggesting the ability of both RVS and AD to trigger deacetylation (S3 Fig). Nevertheless, direct evaluation of SIRT-1 deacetylating activity at the end of the three hour reperfusion interval (Fig 4) together with both functional and morphological analysis of hearts exposed to AD+STN (Figs 1–4) strongly support the involvement of SIRT-1 in AD-mediated cardiac effects. Alternatively, the discrepancy between the low SIRT-1 phosphorylation at the early post-ischemic time-point and the sustained increase of SIRT-1 activity at the end of reperfusion interval might account for a specific biological purpose, as suggested by other studies: for example, in contracting muscle *in vivo* and in cultured C_2_C_12_ cells incubated with AMPK activators, AMPK activation is an early event (s/min), whereas SIRT-1 activation appears to take place much later (4–12 h) [12,61] and would presumably act to sustain the activation of AMPK. Consistent with this, the low levels of phosphorylated LKB1 and AMPK observed in hearts exposed to AD+STN suggest that SIRT-1 inhibition and the subsequent impaired deacetylase ability might interfere with cytoplasmic transfer and subsequent phosphorylation of LKB1, an upstream kinase for AMPK-activation. Similar findings have been described in rat liver, where downregulation of SIRT-1 has been related to an increased lysine acetylation of LKB1, therefore preventing its translocation from the nucleus to the cytoplasm and impairing phosphorylation and activation of AMPK [24].

## Conclusions

**Fig 6:**
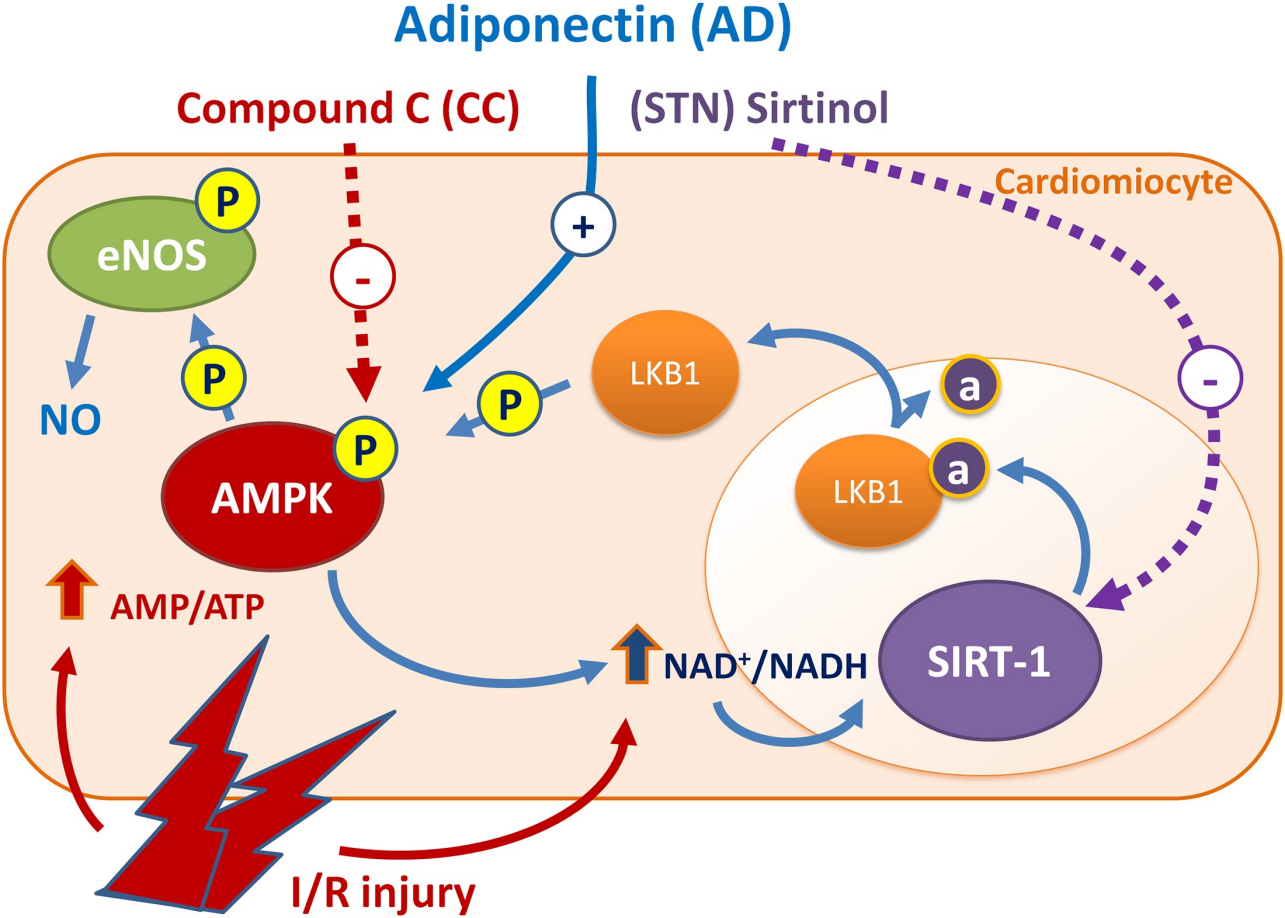
AD preconditioning on isolated rat hearts protects from I/R injury via a signaling pathway involving the AMPK/LKB1/SIRT-1 axis. Phosphorylated AMPK increases eNOS-mediated NO availability, that accounts for the majority of AD cardioprotective effects. By responding to changes in ATP generation/consumption, AMPK plays a key role in modulating energy-consuming anabolic pathways and indirectly participates to SIRT-1 activation through increased NAD^+^ levels and NAD/NADH ratio. In turn, by promoting lysine deacetylation of LKB1 and subsequent translocation from nucleus to cytoplasm, SIRT-1 may sustain the LKB1-mediated activation of AMPK. Combined treatment of AD plus CC (inhibitor of AMPK) or STN (inhibitor of SIRT-1) impair the protective effects of AD preconditioning, suggesting that both AMPK and SIRT-1 are required for AD-mediated cardiovascular protection, and regulate each other activities.

This study provides the first evidence that AD preconditioning on isolated rat hearts protects from I/R injury via a signaling pathway that involves the AMPK/LKB1/SIRT-1 axis (Fig 6*)*. Activation of AMPK is a well known mechanism by which AD phosphorylates and activates eNOS with increased NO availability, that accounts for the majority of AD cardiovascular protective effects. By responding to changes in cellular energy state and ATP generation/consumption, AMPK activation plays a key role in modulating energy-consuming anabolic pathways [62], and can indirectly participate to SIRT-1 activation through increased NAD^+^ levels and NAD/NADH ratio [8,23]. In turn, by promoting lysine deacetylation of LKB1 and subsequent translocation from nucleus to cytoplasm, SIRT-1 may sustain the LKB1-mediated activation of AMPK [63]. Overall, results from our functional, morphological and molecular signaling experiments strongly suggest that both AMPK and SIRT-1 are required for AD-mediated cardiovascular protection, and that both these molecules act as a component of a cycle sharing common intermediated and regulating each other activities. These findings might help to shed light on mechanisms of cardiac protection by circulating endogenous mediators. Undoubtedly, in the prevention of heart damage resulting from acute MI, the strategy described here does not have clinical applicability. On the other hand, for cardiovascular surgery interventions that can be scheduled in advance and require a heart ischemic procedure, this might represent a potential treatment for reperfusion damages prevention. Importantly, in a clinical perspective, our findings highlight the importance of a therapeutic approach aiming at replacing endogenous AD levels in those conditions characterized by low AD concentrations and concomitant increased cardiovascular risk, including acute coronary syndrome [64], obesity-linked complications, type 2 diabetes, dyslipidemia and hypertension [32,65].

## Supporting information

S1 Fig Parameters of left ventricular function and ischemic area in hearts exposed to I/R or vehicle-I/R at the onset of ischemia. Hearts exposed to 30 min ischemia followed by 180 min reperfusion (I/R group) were compared with hearts infused with vehicle alone (3 mL/1 min; vehicle I/R) at the onset of ischemia; both functional parameters (A-D) and the extent of infarct area (E) did not significantly differ between hearts from these two groups. “Vehicle” was modified Krebs’ Henseleit solution (composed of (mmol/l): 118.5 NaCl; 4.7 KCl; 1.2 MgSO_4_; 1.2 KH_2_PO_4_; 1.25 CaCl_2_(H_2_O); 25 NaHCO_3_; 11 glucose) containing DMSO 0.1%.
(PPT)

S2 Fig Left Ventricular Pressure (LVP) values in rat hearts. LVP max, indicating post-ischemic systolic functional recovery, was substantially higher in RSV group (p < 0.01 *vs*. I/R group, at all time-intervals), and slightly increased in AD + STN group vs. respective values in the I/R and AD groups during the second and third hour of reperfusion (p < 0.05 at 90, 120, 180 min).
(PPT)

S3 Fig Protein acetylation levels in heart lysates. Fifteen (15) minutes after reperfusion, hearts from I/R, RSV and AD groups were prepared using lysis buffer, and samples then subjected to immunoblotting (A) or immunoprecipitation (B), according to standard methods. **A**: Samples were separated by 8% SDS-PAGE followed by immunoblotting with anti-acetyl NF-kB p65 ^(Lys310)^ antibody (Cell Signaling). **B.** Lysates were first immunoprecipitated using an antibody against NF-kB p65 (1:50, Cell Signaling), and then subjected to immunoblotting with anti-acetyl NF-kB p65 ^(Lys310)^ antibody (Cell Signaling). A representative blot is shown for triplicate experiments. Qualitatively, acetylation levels on NF-kB p65 (at Lys 310) appear slightly decreased in both RSV and AD groups with respect to I/R group.
(PPT)
