# Supplementary material for: Activation of AMPK/SIRT1 axis is required for adiponectin-mediated preconditioning on myocardial ischemia-reperfusion (I/R) injury in rats
Source: PLoS One. 2019 Jan 17;14(1):e0210654. doi: 10.1371/journal.pone.0210654 (PMC6336234; doi:10.1371/journal.pone.0210654)
Supplement: S1 Fig — Hearts exposed to 30 min ischemia followed by 180 min reperfusion (I/R group) were compared with hearts infused with vehicle alone (3 mL/1 min; vehicle I/R) at the onset of ischemia; both functional parameters (A-D) and the extent of infarct area (E) did not significantly differ between hearts from these two groups. “Vehicle” was modified Krebs’ Henseleit solution (composed of (mmol/l): 118.5 NaCl; 4.7 KCl; 1.2 MgSO4; 1.2 KH2PO4; 1.25 CaCl2(H2O); 25 NaHCO3; 11 glucose) containing DMSO 0.1%. (PPT) [file pone.0210654.s001.ppt]

## Slide 1
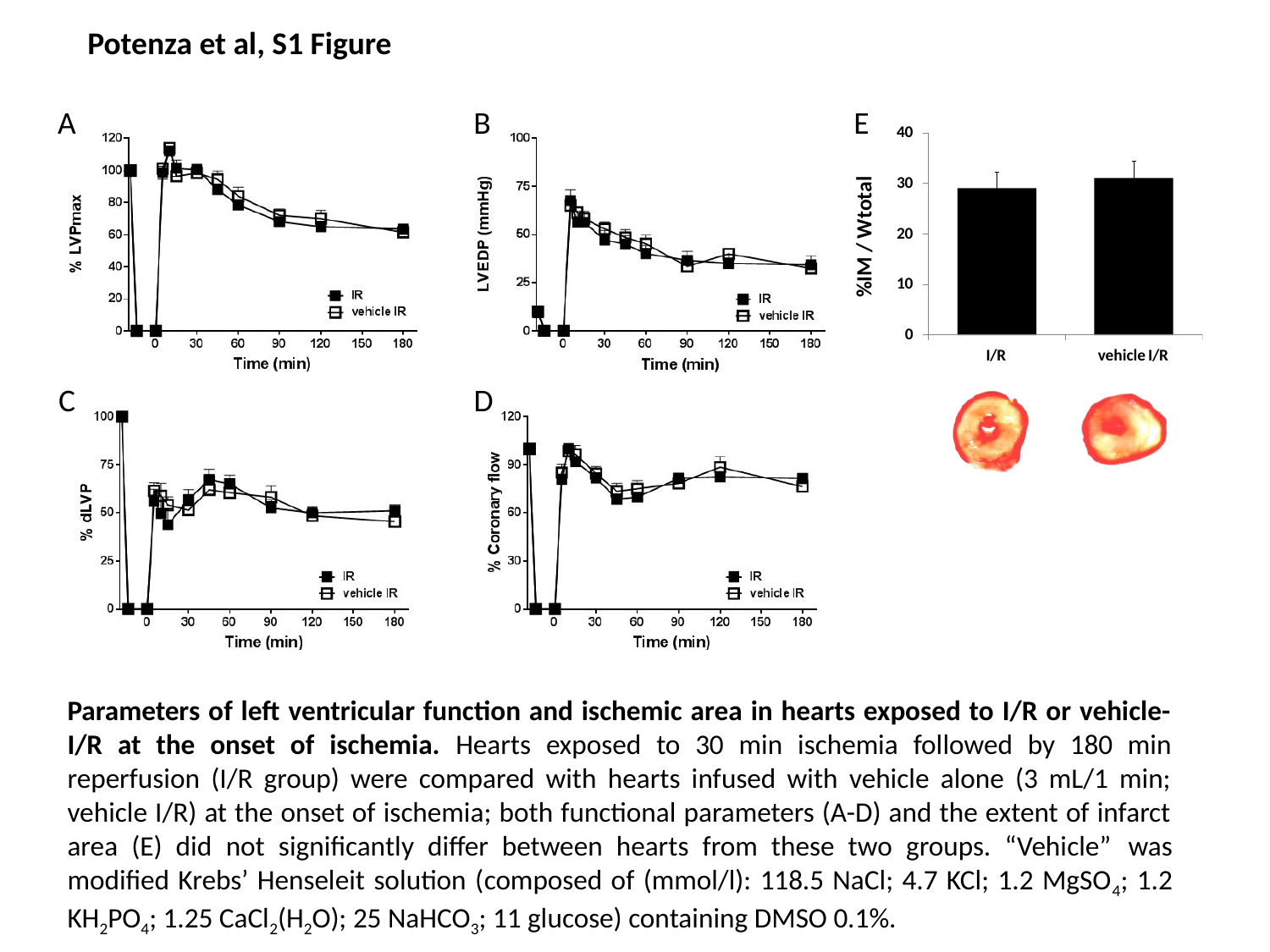

Potenza et al, S1 Figure
A
B
E
C
D
Parameters of left ventricular function and ischemic area in hearts exposed to I/R or vehicle-I/R at the onset of ischemia. Hearts exposed to 30 min ischemia followed by 180 min reperfusion (I/R group) were compared with hearts infused with vehicle alone (3 mL/1 min; vehicle I/R) at the onset of ischemia; both functional parameters (A-D) and the extent of infarct area (E) did not significantly differ between hearts from these two groups. “Vehicle” was modified Krebs’ Henseleit solution (composed of (mmol/l): 118.5 NaCl; 4.7 KCl; 1.2 MgSO4; 1.2 KH2PO4; 1.25 CaCl2(H2O); 25 NaHCO3; 11 glucose) containing DMSO 0.1%.
