# Supplementary material for: Activation of AMPK/SIRT1 axis is required for adiponectin-mediated preconditioning on myocardial ischemia-reperfusion (I/R) injury in rats
Source: PLoS One. 2019 Jan 17;14(1):e0210654. doi: 10.1371/journal.pone.0210654 (PMC6336234; doi:10.1371/journal.pone.0210654)
Supplement: S2 Fig — LVP max, indicating post-ischemic systolic functional recovery, was substantially higher in RSV group (p < 0.01 vs. I/R group, at all time-intervals), and slightly increased in AD + STN group vs. respective values in the I/R and AD groups during the second and third hour of reperfusion (p < 0.05 at 90, 120, 180 min). (PPT) [file pone.0210654.s002.ppt]

## Slide 1
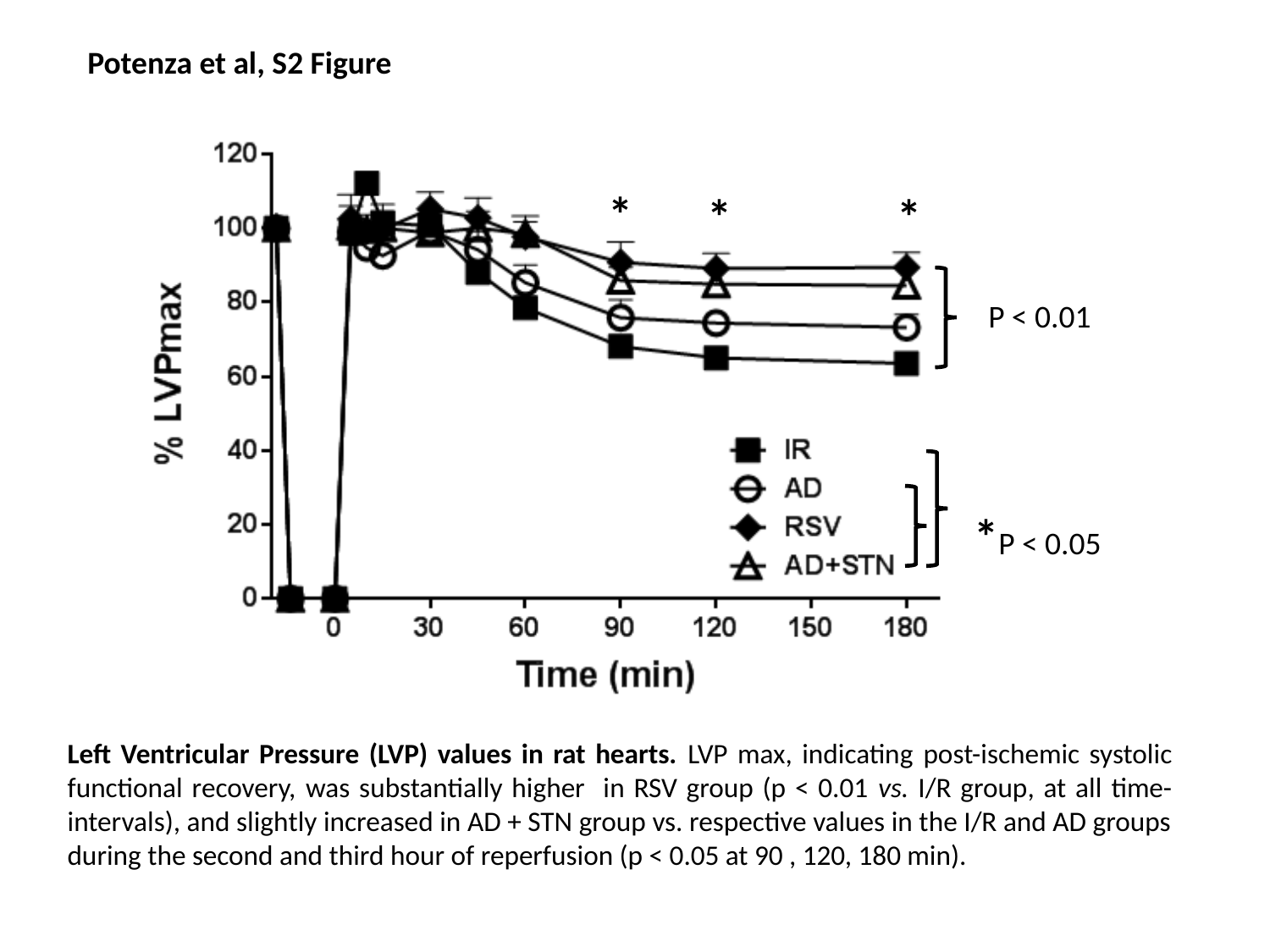

Potenza et al, S2 Figure
*
*
*
P < 0.01
*P < 0.05
Left Ventricular Pressure (LVP) values in rat hearts. LVP max, indicating post-ischemic systolic functional recovery, was substantially higher in RSV group (p < 0.01 vs. I/R group, at all time-intervals), and slightly increased in AD + STN group vs. respective values in the I/R and AD groups during the second and third hour of reperfusion (p < 0.05 at 90 , 120, 180 min).
