# Supplementary material for: Activation of AMPK/SIRT1 axis is required for adiponectin-mediated preconditioning on myocardial ischemia-reperfusion (I/R) injury in rats
Source: PLoS One. 2019 Jan 17;14(1):e0210654. doi: 10.1371/journal.pone.0210654 (PMC6336234; doi:10.1371/journal.pone.0210654)
Supplement: S3 Fig — Fifteen (15) minutes after reperfusion, hearts from I/R, RSV and AD groups were prepared using lysis buffer, and samples then subjected to immunoblotting (A) or immunoprecipitation (B), according to standard methods. A: Samples were separated by 8% SDS-PAGE followed by immunoblotting with anti-acetyl NF-kB p65 (Lys310) antibody (Cell Signaling). B. Lysates were first immunoprecipitated using an antibody against NF-kB p65 (1:50, Cell Signaling), and then subjected to immunoblotting with anti-acetyl NF-kB p65 (Lys310) antibody (Cell Signaling). A representative blot is shown for triplicate experiments. Qualitatively, acetylation levels on NF-kB p65 (at Lys 310) appear slightly decreased in both RSV and AD groups with respect to I/R group. (PPT) [file pone.0210654.s003.ppt]

## Slide 1
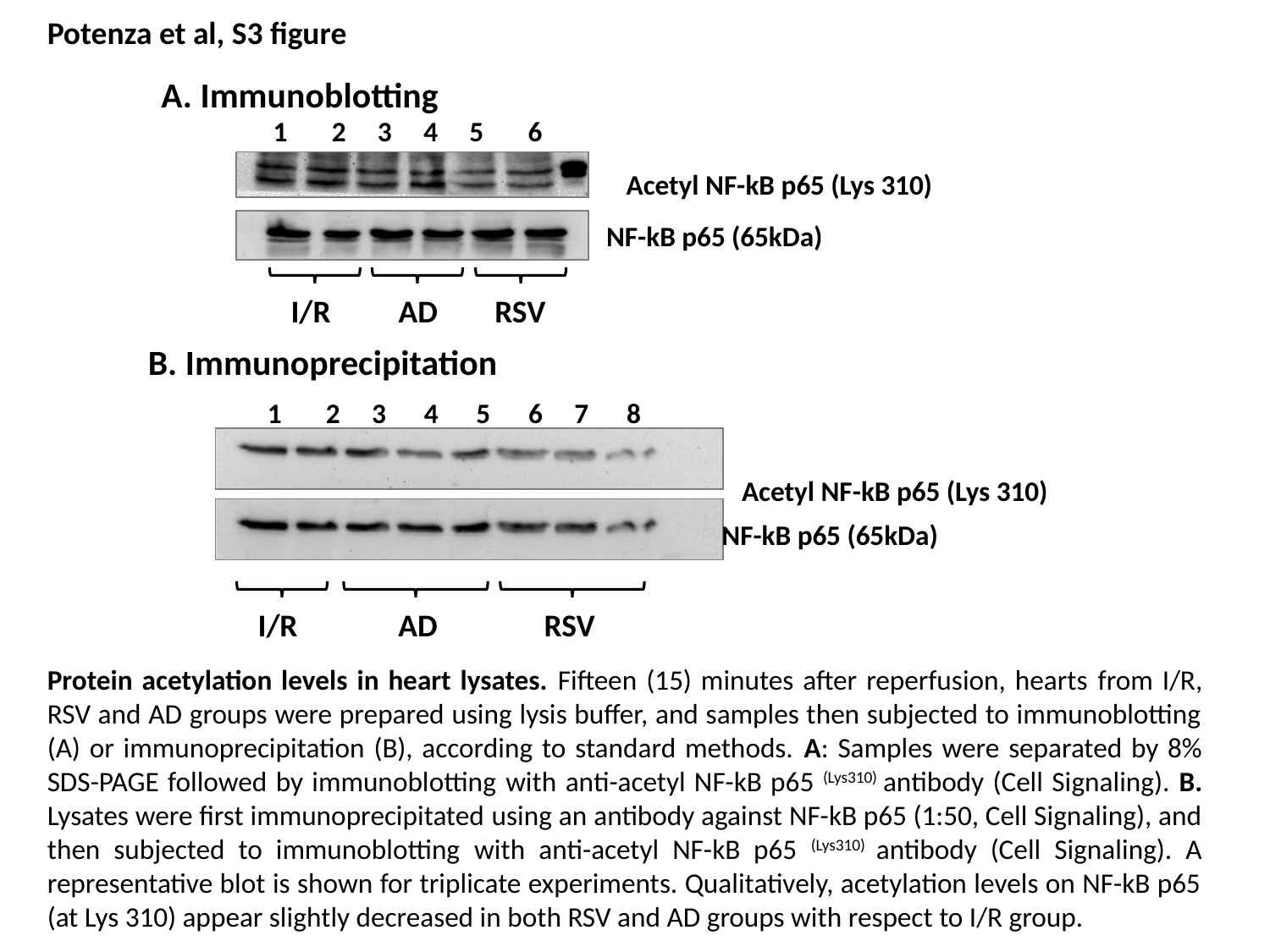

Potenza et al, S3 figure
A. Immunoblotting
1 2 3 4 5 6
Acetyl NF-kB p65 (Lys 310)
NF-kB p65 (65kDa)
I/R
AD
RSV
B. Immunoprecipitation
1 2 3 4 5 6 7 8
Acetyl NF-kB p65 (Lys 310)
NF-kB p65 (65kDa)
I/R
AD
RSV
Protein acetylation levels in heart lysates. Fifteen (15) minutes after reperfusion, hearts from I/R, RSV and AD groups were prepared using lysis buffer, and samples then subjected to immunoblotting (A) or immunoprecipitation (B), according to standard methods. A: Samples were separated by 8% SDS-PAGE followed by immunoblotting with anti-acetyl NF-kB p65 (Lys310) antibody (Cell Signaling). B. Lysates were first immunoprecipitated using an antibody against NF-kB p65 (1:50, Cell Signaling), and then subjected to immunoblotting with anti-acetyl NF-kB p65 (Lys310) antibody (Cell Signaling). A representative blot is shown for triplicate experiments. Qualitatively, acetylation levels on NF-kB p65 (at Lys 310) appear slightly decreased in both RSV and AD groups with respect to I/R group.
